# Alleviating the medical strain: a triage method via cross-domain text classification

**DOI:** 10.3389/fncom.2024.1468519

**Published:** 2024-12-18

**Authors:** Xiao Xiao, Shuqin Wang, Feng Jiang, Tingyue Qi, Wei Wang

**Affiliations:** ^1^Department of Ultrasound, The Affiliated Hospital of Yangzhou University, Yangzhou University, Yangzhou, Jiangsu, China; ^2^Department of Information Engineering, Yangzhou University, Yangzhou, Jiangsu, China; ^3^Department of Ultrasound, The First Affiliated Hospital of Wannan Medical College, Wuhu, Anhui, China; ^4^Department of Radiology, The Affiliated Hospital of Yangzhou University, Yangzhou University, Yangzhou, Jiangsu, China

**Keywords:** medical triage, cross-domain text classification, prompt-tuning, few-shot, domain adaptation

## Abstract

It is a universal phenomenon for patients who do not know which clinical department to register in large general hospitals. Although triage nurses can help patients, due to the larger number of patients, they have to stand in a queue for minutes to consult. Recently, there have already been some efforts to devote deep-learning techniques or pre-trained language models (PLMs) to triage recommendations. However, these methods may suffer two main limitations: (1) These methods typically require a certain amount of labeled or unlabeled data for model training, which are not always accessible and costly to acquire. (2) These methods have not taken into account the distortion of semantic feature structure and the loss of category discriminability in the model training. To overcome these limitations, in this study, we propose a cross-domain text classification method based on prompt-tuning, which can classify patients' questions or texts about their symptoms into several given categories to give suggestions on which kind of consulting room patients could choose. Specifically, first, different prompt templates are manually crafted based on various data contents, embedding source domain information into the prompt templates to generate another text with similar semantic feature structures for performing classification tasks. Then, five different strategies are employed to expand the label word space for modifying prompts, and the integration of these strategies is used as the final verbalizer. The extensive experiments on Chinese Triage datasets demonstrate that our method achieved state-of-the-art performance.

## 1 Introduction

Nowadays, clinical departments in general hospitals have the characteristics of wide distribution and high complexity. As the first step of patients seeking medical treatment, triage plays a significant role in clinic work (Haas et al., 2014; van der Linden et al., 2016). However, due to the lack of medical knowledge, many patients often do not know how to choose a department when they first see a doctor, which leads to the phenomenon of patients choosing the wrong departments. Moreover, because of the large number of patients, they have to wait in line for minutes to consult. It causes existing triage services to not fully meet the needs of patients. The Internet can break geographical and spatial restrictions, change the traditional way of registration, and provide patients with a fair and reasonable utilization of high-quality medical service resources. To a large extent, it can reduce the workload of triage nurses, alleviate the problem of registration difficulty for the patients, and improve the medical experience. Many general hospitals have used the intelligent guidance system by providing clinical departments for patients to choose from. It fails to make full use of data for mining and even cannot provide the correct data reference for future statistical analysis. Therefore, it is important to explore a method for medical triage, i.e., clinical department recommendation (Veladas et al., 2021).

Recently, there have already been some efforts to devote deep-learning techniques or pre-trained language models (PLMs) to triage recommendations. Some methods utilize deep schemes to learn the representative features and achieve superior performance for medical triage. For example, Gao X. et al. (2021) applied word2vec to generate a word vector of Chinese patient symptom descriptions, and the performance of mainstream deep neural networks, such as CNN, RNN, and RCNN, is analyzed on triage recommendation. Recently, with the widespread use of PLM models in various downstream tasks, fine-tuning models allow the upstream pre-trained knowledge to be fully applied to downstream subtasks including medical triage (Howard and Ruder, 2018). For example, BERT encodes the medical Question Answering datasets and is modified with additional components specified to medical triage (Wang et al., 2021).

Despite these advances, two main limitations prevent the further development of these methods: The first challenge pertains to a certain amount of labeled or unlabeled data for model training. Not only do the deep neural networks require large-scale labeled training datasets, but the recent success of PLMs also hinges on fine-tuning them on large amounts of labeled data for the downstream tasks, which are typically expensive to acquire and difficult to use for real applications in medical triage. The second challenge is the semantic information loss in model training. On the one hand, the significant gap between the objective forms in pre-training and fine-tuning limits the advantage of rich knowledge in PLMs. On the other hand, the existing methods have not taken into account the distortion of semantic feature structure and the loss of category discriminability in the model training. The semantic information loss here refers to the loss that incurred by different data distributions in the source and target domains. For example, the source domain is a symptomatic description about surgery, where the patient experiences sudden severe chest pain accompanied by difficulty breathing, and the pain cannot be relieved. The target domain is the description related to internal medicine, where the patient experiences sudden abdominal pain lasting for ~12 h, with an unstable pain location, accompanied by vomiting and mild fever, and indicates recent instances of eating unclean food. The data distribution varies greatly between domains; thus, semantic information loss can be incurred.

**Figure 1:**
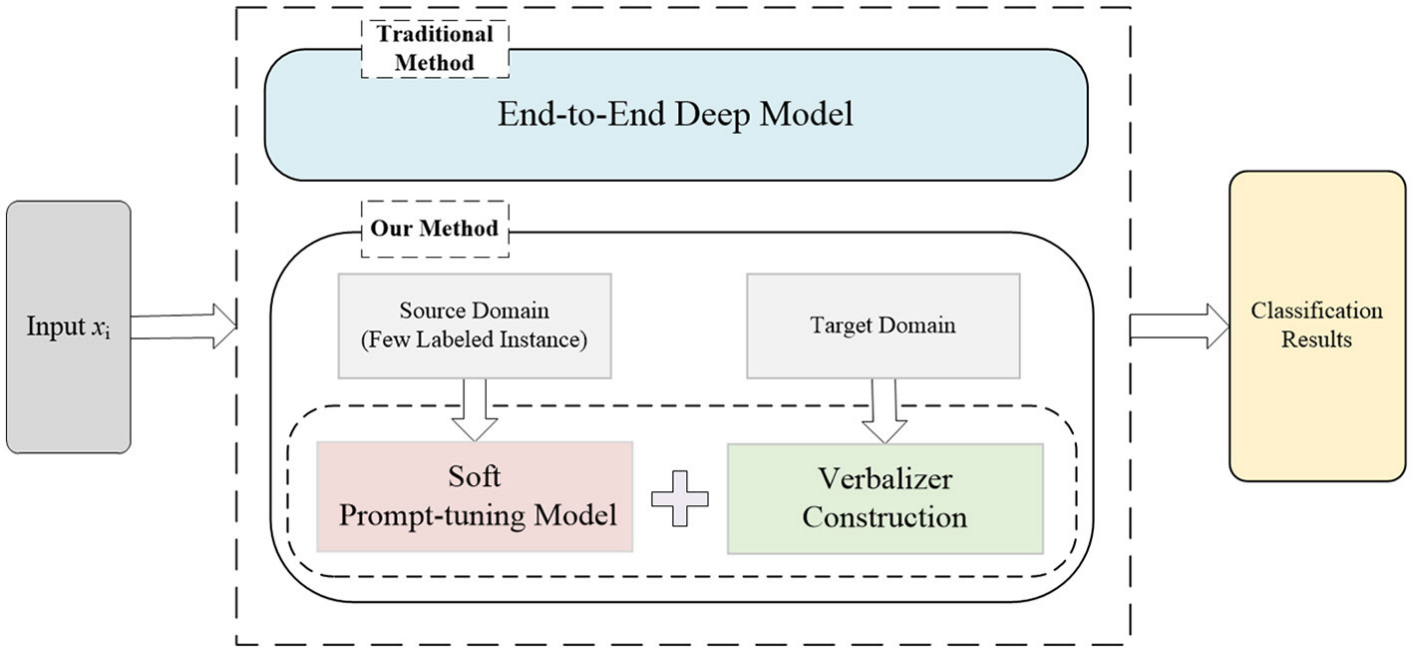
The motivation of our method. The traditional medical triage method trained an end-to-end deep model, which is not only time-consuming and labor-intensive but also requires large-scale labeled training data. In contrast, our method stems from the recent success of prompt-tuning, which is trained in the source domain with few labeled instances. The verbalizer is then constructed to preserve the semantic information in the domain.

To address these limitations, in this study, we propose a cross-domain text classification method based on prompt-tuning for medical triage, which aims to effectively alleviate the medical strain. We compared our method with the traditional method as shown in the Figure 1. Our method can classify patients' questions or texts about their symptoms into several given categories to give suggestions on which kind of consulting room patients could choose. (1) For *Challenge 1*, we propose a prompt-tuning method for medical triage. Prompt-tuning fills the input statements into the natural language template and adapts the masked model, which formalized regarded the downstream NLP task as cloze-style tasks (Ding et al., 2021b; Liu et al., 2023). Compared with the previous deep-learning and fine-tuning PLMs approaches, no additional neural layer is needed in prompt-tuning, and excellent performance has been achieved even in the scenario of few-shot learning. (2) For *Challenge 2*, we propose a cross-domain text classification method. Domain-specific templates are used in prompt-tuning, combining templates with domain information to preserve the semantic information of instances in the domain, which can solve the problem of aligning global feature representations between domains at the cost of losing semantic information in existing methods. For example, a template is manually set for the source domain sentence, the target domain sentence is embedded into the template, and prompting-tuning will make predictions based on the probability of filling label words. Extensive experiments on Chinese Triage datasets demonstrate that the proposed method outperforms the state-of-the-art methods. In summary, the contributions of this study are as follows:

- We propose a novel prompt-tuning method for medical triage, and cross-domain text classification is introduced to alleviate the medical strain. Our method can fully utilize the knowledge of pre-trained language models and achieve state-of-the-art results.
- Our method can align global feature representations between domains without the cost of losing semantic information.
- In contrast to the existing methods that require amounts of training data, our method can achieve state-of-the-art performance with only a small number of labeled instances.
- We experimentally show that our method is more robust and effective than SOTA baselines on Chinese benchmark datasets for medical triage.

## 2 Related work

### 2.1 Medical triage

Medical triage aims to recommend clinical departments automatically, which helps patients to complete self-diagnosis, improve the efficiency of triage, and reduce the work intensity of triage nurses. Nowadays, the number of patients visiting the hospital has been increasing. In many cases, patients unfamiliar with symptoms and diseases would have difficulty registering putting huge pressure on the triage system in the hospital. This situation is common among many hospitals in China. Even though hospitals have set up many triage tables, compared with the number of patients, the shortage of experienced staff at triage desks leads to heavy workloads and low efficiency, resulting in poor service and patient dissatisfaction. Moreover, some staff are not professional enough for various diseases and provide the wrong guidance to patients. Some solutions are raised to solve the problem. For example, some hospitals launched APP for reservation, an application called Hospital Registration APP created by Nanjing University of Science and Technology (Yaqi, 2018). In addition, some researchers have developed medical chatbot frameworks, such as the Application effect of intelligent (Bao et al., 2020), but they are not specified for triage use. Medical triage can be framed as a classification problem: given definitions and descriptions of clinical departments and symptomatic information from the patients, determine which department is the patient to choose (Gao X. et al., 2021; Wang et al., 2021).

The research paradigm of medical triage evolved from early feature engineering-based methods to neural networks and, more recently, into pre-trained language models such as BERT, which also show superiority in this task. Traditional medical triage methods mainly use feature engineering to capture text features (Schuetz et al., 2013), such as logistic regression, support vector machine, and decision tree can be applied for triage recommendation (Fernandes et al., 2020). However, in the field of medicine, text features need to be recognized by professional doctors, which leads to a very expensive and difficult process that requires a lot of time and expertise.

In the past few years, deep neural networks have shown remarkable performance in various tasks including medical triage due to their ability to learn more abstract and higher-level feature representations. For example, Kim (2014) proposed TextCNN with little hyper-parameter tuning and static vectors for cross-domain text classification, which generated the data confusion domain discriminator to minimize deviations between different domains. Shi et al. (2023) proposed a two-layer BiLSTM neural network to categorize medical complaints, enabling intelligent triage for telehealth services. In the method, a 1.3 GB medical dataset comprising 200,000 records, each including medical history, physical examination data, and other pertinent information, is gathered for embedding learning. Lai et al. (2015) used recurrent convolutional neural network (RCNN) to capture contextual information as far as possible, and the accuracy of classification results has been significantly improved. Gao X. et al. (2021) applied word2vec to generate a word vector of Chinese patient symptom descriptions, and the performance of mainstream deep neural networks, such as CNN, RNN, and RCNN, is analyzed on triage recommendation.

More recently, pre-trained language models (PLMs) such as GPT, BERT, RoBERTa, and T5 have emerged as a powerful instrument for language understanding and generation, which can capture syntactic (Goldberg, 2019), semantic (Ma et al., 2019), and structural (Jawahar et al., 2019) information. Fine-tuning PLMs with task-specific headers has become a major practice and has demonstrated strong performance in various NLP downstream tasks, including machine translation (Zhu et al., 2020), text classification (Minaee et al., 2021), question answering (Adiwardana et al., 2020), lexical simplification (Qiang et al., 2020), and domain adaptation (El Mekki et al., 2022). For example, Wang et al. (2021) developed a text classification model called TriageBert and built a web triage system. In the method, two different data pre-processing strategies are used to generate two datasets with different sizes and develop two models, TriageBertS and TriageBertL (Wang et al., 2021). Then, these two models are modified with additional components specified for medical triage. Wang et al. (2023) proposed an intelligent triage system combining the BERT model and the Triage Priority (TP) method, which optimizes multi-label medical text classification in Neurology by transforming the multi-label problem into a single-label problem while utilizing a composite loss function based on cost-sensitive learning to solve the data imbalance problem. However, the significant gap of objective forms in pre-training and fine-tuning restricted taking full advantage of knowledge in PLMs. In addition, most of these methods have not taken into account the distortion of semantic feature structure and the loss of category discriminability in the model training.

The use of Artificial Intelligence (AI) in healthcare triage has grown significantly in recent years, especially when responding to the pressures of emergencies in the healthcare system, where AI shows greater potential. Paslı et al. (2024) evaluated the accuracy of artificial intelligence (ChatGPT) in emergency department triage decisions, demonstrating the effectiveness of AI tools in triaging patients in the emergency department. Meng Sr and Tang Sr (2024) explored the real-world performance of large-scale language models in emergency department chest pain triage, confirming the potential of LLMs to enhance emergency medical diagnosis, especially in resource-limited settings. While artificial intelligence models exhibit many advantages, they also have some limitations. For example, these models rely on data input provided by humans and are unable to memorize chats after a certain number of words. Therefore, AI models are not a complete replacement for specialized triage teams and should be viewed more as an adjunct to support the triage process rather than a standalone alternative.

Therefore, based on the above work, we propose a medical triage method by cross-domain text classification based on prompt-tuning.

### 2.2 Prompt-tuning

More recently, prompt-tuning (Liu et al., 2023) effectively utilizes pre-training information by transforming downstream tasks into cloze-style forms.

Earlier works mainly rely on hand-engineered discrete prompts (Han et al., 2022) that remain constant during training. However, designing manual prompts suitable for downstream tasks is time-consuming and labor-intensive. To address this problem, a series of methods have been explored for automatically generating prompts. For example, Jiang et al. (2020) proposed an automatic prompt-generation method based on mining and paraphrasing. In the method, the relation extraction approaches are used as mining-based methods, and paraphrasing-based methods aim to paraphrase seed prompts into semantically similar expressions. Finally, the lightweight ensemble method is introduced to combine different prompts together. Liu et al. (2021) proposed a method to transform template construction into a continuous parameter optimization problem, called P-Tuning. Given discrete prompts as input, P-Tuning connects continuous prompt embedding with discrete prompt tokens and inputs them into the pre-trained language model. The continuous prompts are updated through back-propagation to optimize task objectives. Zhu et al. (2024) proposed a soft template generation method for short text classification, which employed five different strategies to expand the label word space from the open knowledge base.

In prompt-tuning, verbalizer refers to a mapping from labeled words to categories, which is an effective strategy to improve model performance (Gao T. et al., 2021). Although manually constructed verbalizers perform well in downstream NLP tasks, they are influenced by prior knowledge and can lead to omissions and biases in knowledge augmentation. Recently, a range of automatic verbalizer construction methods has been proposed (Schick et al., 2020). For example, Hu et al. (2021) introduce external knowledge and perform refinement to filter out words containing noise. In the method, the open knowledge graph, such as Related Words, is introduced to retrieve related works to the name of each topic. Then, the verbalizer refinement is further conducted by retaining high-quality words and removing low-relevance words. Wei et al. (2022) proposed a prototype-cued verbalizer approach to gain knowledge from a pre-trained language model. The prototypical network is utilized to generate embeddings for different labels in the feature space, and contrastive learning is introduced to devise different objective functions for optimizing the model. Zhu et al. (2023) proposed an automatic verbalizer construction method for short text classification, which takes both the short text itself and the class name into consideration during expanding label word space. Moreover, in the field of clickbait detection, Wu et al. (2023) proposed the Part-of-speech Enhanced Prompt Learning method (PEPL) for detecting clickbaits in Chinese social media.

## 3 Methodology

### 3.1 Motivation

Despite the advances of existing deep learning and PLMs for medical triage, the requirement of training data and semantic information loss still prevent the further development of these approaches. In this study, we propose a cross-domain text classification method based on prompt-tuning for medical triage, which aims to effectively alleviate the medical strain. Our method can combine information from different domains with designed templates based on prompt-tuning, which can fully preserve the semantic information of data samples in the domain and align the global feature representations between different domains through the rich semantic features of PLMs. Our method can solve the problem of aligning global feature representations between domains at the cost of losing semantic information in deep neural networks and PLMs.

The basic idea of our method is to use prompt-tuning to convert text input sequences from different domains into cloze tasks with the same template when the data distribution in the source and target domains is different. The mask language model is used to minimize the gap between the source and target domains by leveraging the rich features of PLMs. In addition, our method can adapt to the upstream PLMs through downstream classification tasks based on prompt-tuning, which trains the prompt model with only a small number of samples and classifies and predicts unlabeled data based on the trained prompt model. Our method can solve the problem of requiring a large amount of data for training models in existing methods and achieves a good medical triage performance through the prediction of the masked language model with only a small number of labeled samples.

### 3.2 Overall framework

**Figure 2:**
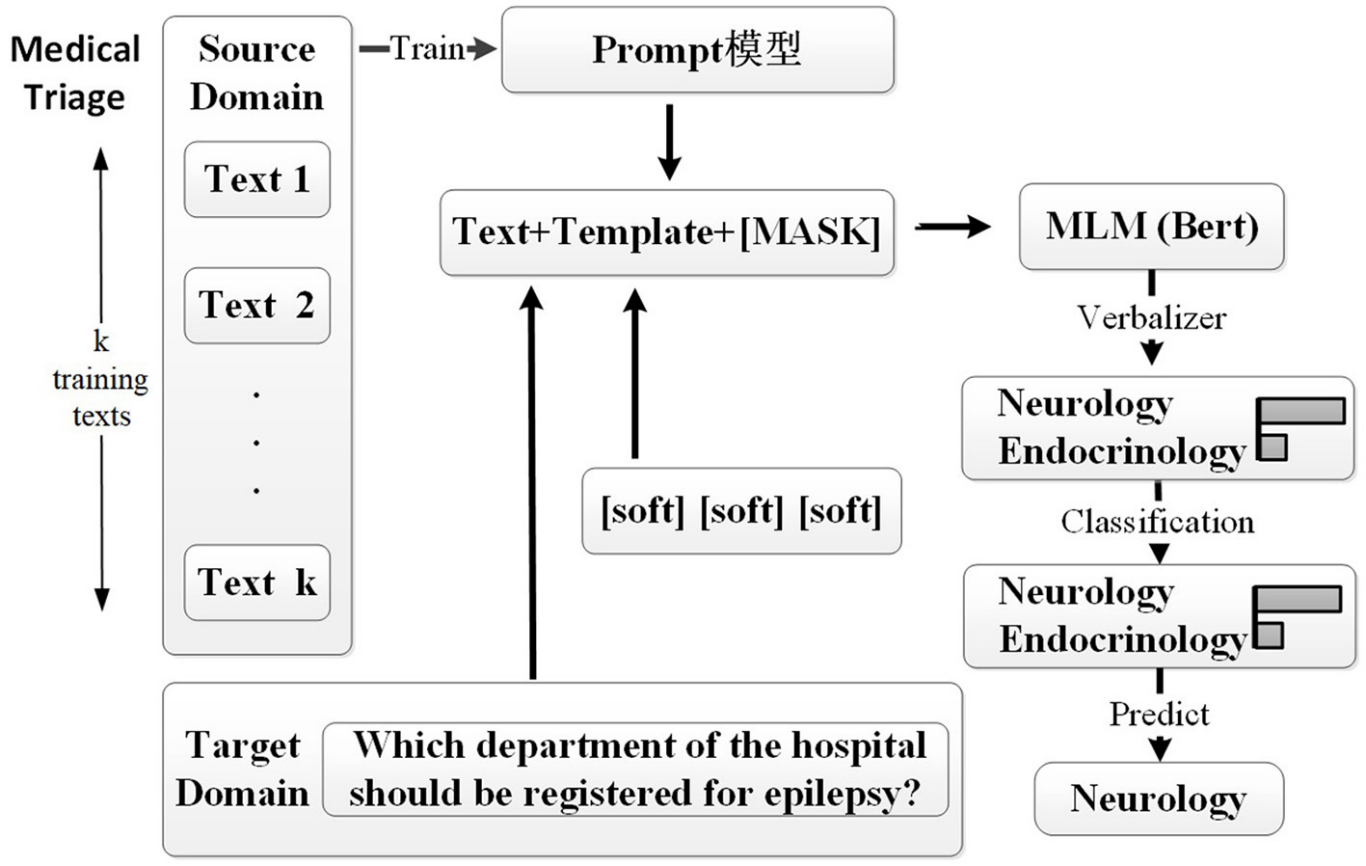
This framework outlines our approach. The source domain consists of labeled medical inquiry data, while the target domain comprises patient inquiry data lacking explicit department labels. Initially, the prompt-tuning model is trained with a limited number of examples from the source domain. For instance, text-i represents the *i*th text data; for example, text-1: “What should I do if my child has a fever?” is labeled as “Pediatrics.” Each training text is converted into a defined soft template format and fed into the model. The masked language model (MLM) predicts the words at the [MASK] position based on the context and maps them to specific medical departments through the verbalizer. Subsequently, target domain data are embedded in a soft template, and the model classifies the text. Finally, the predicted tags are mapped to the target domain sentence categories, enabling the classification of target domain sentences. For example, “Which department of the hospital should be registered for epilepsy?” is predicted as “Neurology.”

The overall framework of our proposed medical triage method is illustrated in Figure 2. Specifically, the medical triage can be seen as a classification task, which is first converted into a natural language sequence in the cloze-task form by template, and only a small number of labeled source domain data samples are used for training. Then, the sentences in the target domain are embedded into the same template, and the pre-trained language model is modeled for the mask language model. Finally, the result is predicted by the label words and mapped to the label category of the sentences in the target domain.

Given the source domain *D*_*S*_ with labeled data as $X_{S}=\left\{x_{i}^{\left(s\right)},y_{i}^{\left(s\right)}\right\}{|}_{i=1}^{n_{s}}$, the target domain *D*_*T*_ with unlabeled data as $X_{T}=\left\{x_{i}^{\left(t\right)}\right\}{|}_{i=1}^{n_{t}}$, where *n*_*s*_ and *n*_*t*_ are the number of instances in source and target domain, respectively, $x_{i}^{\left(s\right)}\in {ℜ}^{m\times 1}$ and $x_{i}^{\left(t\right)}\in {ℜ}^{m\times 1}$, $y_{i}^{\left(s\right)}\in \left\{1,2,...,c\right\}$. The goal of cross-domain text classification for medical triage is to train a classifier in data of the source domain to make precise predictions on the target domain.

### 3.3 Cross-domain text classification method based on prompt-tuning for medical triage

In our method based on prompt-tuning, input statements are first embedded into a natural language template, which is trained on a few labeled data in the source domain. Then, the trained model is applied to the target domain to perform the classification task as medical triage.

In the template design in prompt-tuning, there are two main methods, hand-crafted and auto-generated templates, that are employed to formalize downstream NLP tasks into cloze-style filling tasks. Due to the incorporation of rich domain expertise, manual templates have been proven to be more competitive and superior compared to automatically generated templates (Gao et al., 2020). To make the prompt context fluent and instructive, we set different templates for separate dataset tasks, and the detailed experimental results are shown in Section 4.5. For instance, given the input sentence, *x* as “Which department of the hospital should be registered for epilepsy?”, prompt-tuning with a hand-crafted template wraps it into “x_p_ = [CLS]x, this text is about [MASK].” The [CLS] is placed at the beginning of a sentence to represent the vector obtained through the pre-training language model for subsequent classification tasks. In the experiments, it was learned through the Bert model. The [*MASK*] is to be filled in the label words, and the probability of different topic words, such as “Neurology” or “Endocrinology,” is then calculated to fill the “[MASK]” token.

In prompt-tuning, verbalizer refers to mapping labeled words to their corresponding categories, which has been proven to be an effective method for improving downstream task performance (Hu et al., 2021). In our method, we incorporate three additional strategies aimed at expanding the label words originating from the input sentence. These strategies not only mitigate the impact of noise in the expanded label words but also enhance efficiency by reducing runtime. Each strategy captures distinct aspects of the expanded word's characteristics, all of which are incorporated into the final verbalizer. The specifics of these three strategies are outlined below:

BERT prediction: to leverage the rich knowledge in the PLMs and obtain the probability distribution of the [MASK] token in the template, we sort all the words according to their probabilities and select the top *N* words.

FastText similarity: we compute the semantic similarity between the FastText vectors of each class label word and the extended label words. We also select the top *N* words with higher similarity.

Context information: taking the context information into consideration, the expanded words should consider the surrounding words before and after the [MASK] token. We replace the [MASK] with the expanded word and mask one of the words around it from front to back. We then feed it into BERT to calculate the cross-entropy loss. Similar to other strategies, the top *N* terms are selected based on this process.

Finally, the union of expanded words selected by three strategies is used as the final verbalizer. Notably, the *N* = 15 is adopted in the experiments.

### 3.4 Medical triage recommendation

Once the verbalizer has been constructed, as each word in the final verbalizer is assumed to have an equal contribution to the prediction, we calculate *g* by taking the average of the predicted scores for classification. *g* can be calculated as


(1) $$\begin{array}{l} g=\frac{1}{\left|L\right|}\overset{\left|L\right|}{\underset{i=1}{\sum }}p\left(\left[\text{MASK}\right]=y_{i}|T\left(x\right)\right) \end{array}$$

where |L| represents the total number of labels, *y*_*i*_ is the *i*th label, *T*(*x*) is the soft template for instance *x* in the target domain, and *p*([MASK] = *y*_*i*_|*T*(*x*)) is the predicted probability of label *y*_*i*_ for instance *x*.

## 4 Experiments

### 4.1 Datasets

Medical Q&A datasets^1^: this dataset is publicly available and comprises 792,099 question–answer pairs spanning six distinct departmental domains, each encompassing various sub-disciplines. In our experiments, we focused on four specific departmental domains: Pediatrics, Internal medicine (Internal), Surgery, and Oncology. We established four distinct cross-departmental domain tasks, for which we selected 1,000 positive and 1,000 negative examples from both the source and target domains in each task. The data distribution between the source and target domains varies due to the differing sub-disciplines within each department.

### 4.2 Baseline methods and implementation details

#### 4.2.1 Baseline methods

- TextCNN (Kim, 2014) The method uses source domain data to train a convolutional neural network and then applies the trained model to the target domain for cross-domain classification tasks.
- SDA (Glorot et al., 2011) This method aims to extract feature representations in the source domain, train a classifier on these features, and then apply the trained model to the target domain to perform classification.
- LLaMA3 (Touvron et al., 2023): A highly efficient large-scale language model created by Meta, optimized for low-resource environments. It achieves strong performance in natural language reasoning and generation tasks while reducing computational costs by minimizing the number of parameters.
- Mistral (Jiang et al., 2023): A cutting-edge large-scale language model developed by the Mistral AI team, known for its outstanding computational efficiency and generative abilities. It delivers superior performance, especially in multi-modal tasks, when compared to other models in its class.
- Prompt (Ding et al., 2021a) This method is a technique for guiding natural language processing models to generate or understand text by providing specific contexts or instructions, enabling the models to perform tasks more accurately.

#### 4.2.2 Implementation details

In this study, given that TextCNN and SDA models require substantial amounts of data for effective training, all source domain samples from the dataset were utilized to train these models. To ensure experimental fairness, Prompt and our proposed method maintained consistent parameter settings. During training, we set the dropout rate to 0.5, the learning rate to 3e-5, the batch size to 32, and the number of epochs to 5, ensuring training robustness and result stability. For few-shot learning, we used 20 samples (shots) and experimented with three random seeds, averaging the results to ensure reliability. In the experiments of TextCNN method, for the four tasks, the sample sizes of source domain data are 1,200, 1,600, and 1,800 samples, respectively. For the experiments of the SDA, LLaMA, and Mistral method, the sample sizes of the source domain data for the four tasks are 1,200, 1,600, and 2,000 samples, respectively. For the experiments of Prompt and our proposed method, the source domain data sample sizes for the four tasks are 2, 10, and 20 samples, respectively.

All experiments were conducted on a high-performance server equipped with an NVIDIA GeForce RTX 3090 Founders Edition GPU, an Intel (R) Core (TM) i9-10980XE CPU operating at 3.00 GHz, and 125 GB of RAM. The experimental environment utilized Python 3.6, PyTorch 1.10.1, and OpenPrompt 0.1.1, providing a robust and efficient setup for validating various approaches in cross-domain tasks.

These meticulously controlled experimental conditions allowed us to fairly evaluate the performance of different methods and draw reliable conclusions, which will inform future research and applications.

### 4.3 Experimental results

To ensure the accuracy of the experimental results, all experiments were conducted three times and averaged. Table 1 shows the experimental results for each task. Based on the experimental results, we have the following observations:

**Table 1:** Experimental results (accuracy %) of our method with comparison methods.

**Tasks**	**Method**	**Number of source domain samples**		
		**1,200/1,200/2**	**1,600/1,600/10**	**1,800/,2000/20**
Oncology → Internal	TextCNN	56.58	59.73	67.20
	SDA	64.79	69.11	77.07
	LLaMA	76.23	76.40	77.00
	Mistral	73.78	74.46	74.73
	Prompt	66.65	71.50	80.20
	Ours	**82.65**	**90.03**	**95.47**
Surgery → Oncology	TextCNN	**71.42**	72.33	75.05
	SDA	57.88	59.93	64.58
	LLaMA	60.81	61.06	61.75
	Mistral	66.07	66.75	67.18
	Prompt	62.87	**73.13**	76.60
	Ours	59.50	68.90	**76.87**
Pediatric → Internal	TextCNN	65.37	70.22	77.60
	SDA	75.92	82.79	87.38
	LLaMA	82.39	83.44	84.14
	Mistral	84.44	86.13	86.21
	Prompt	88.77	94.13	94.62
	Ours	**85.15**	**94.57**	**95.28**
Pediatric → Surgery	TextCNN	85.29	86.98	89.78
	SDA	73.97	78.84	86.33
	LLaMA	79.69	81.93	82.25
	Mistral	85.76	86.32	86.89
	Prompt	85.72	**94.17**	94.30
	Ours	**90.02**	92.45	**94.82**

The bolded result indicates the best result.

(1) In the experiments, the number of source domain samples varied across different methods. However, as the number of source domain samples increased, the performance of most models consistently improved. This indicates that the number of labeled source domain samples positively impacts the experimental results.

(2) Deep-learning methods, such as TextCNN and SDA, rely on large amounts of training data to learn robust feature representations, achieving strong performance in cross-domain text categorization tasks. However, when training data are limited or there is a domain mismatch, the performance of these methods can degrade significantly.

(3) Large models have the ability to capture broad semantic and contextual information, and they typically perform well across various downstream tasks. They can be fine-tuned with a small amount of data to adapt to dichotomous classification tasks in different domains quickly. However, the training of large models often requires high computational resources and still relies on a certain amount of labeled data for fine-tuning. When the training data are small, it may face the risk of overfitting.

(4) The prompt-tuning method particularly excels in few-shot learning. Because the pre-trained model has already acquired much knowledge, it can achieve good results with fewer training samples through clever prompt design. In contrast, TextCNN and SDA require more training data to learn effective feature representations. Traditional prompt learning methods utilize only a small amount of labeled data from the source domain or ignore the data from the target domain when constructing prompts. In contrast, our method fully leverages the data from both the source and target domains by combining multiple strategies to reduce bias between the two. This approach significantly improves the accuracy and robustness of cross-domain text categorization.

(5) Manual templates in prompt learning usually consist of fixed words or phrases, which may make it difficult to express complex task requirements fully. Our approach replaces hard templates with soft templates based on prompt learning. The soft-prompt adaptation method can automatically generate prompt words based on specific task requirements and capture task-specific features more effectively.

(6) Overall, our approach achieves outstanding results on most tasks. In both the source and target domains, we employ multiple expansion strategies to capture different levels and types of information. By combining these strategies, we can extend the labeled words more comprehensively and reduce dependence on specific domains or sources, thus significantly improving the accuracy and robustness of cross-domain text categorization. By using soft templates to construct label prediction prompts, we reduce the domain bias introduced by manual templates and enhance the model's generalization ability. Consequently, our method is more effective at handling cross-domain classification problems compared to other methods.

### 4.4 Influence of templates

Templates are a central part of prompt-tuning, and the construction of templates has a significant impact on model performance. In this section, we will compare the effects of hard and soft templates through a series of experiments. We uniformly configured the soft templates as *x*, [*soft*][*soft*][*soft*][*MASK*] and conducted experiments using four distinct hand-designed templates, detailed in the accompanying Table 2. Given that the dataset used in these experiments is Chinese, we translated the English templates directly into Chinese before proceeding with the experiments.

**Table 2:** Different templates on datasets.

**ID**	**Templats**
0	[MASK] department: X
1	X, The text is about [MASK]
2	X, This text belongs to the [MASK]
3	This text belongs to the [MASK], X

The experimental results are shown in Table 3. The results show that the soft template exhibits higher accuracy overall. Upon analysis, we found that the soft template can dynamically adapt to changes in the input data, whereas the hard template is fixed. This dynamic adaptation makes the soft templates more flexible in dealing with data and tasks in different domains, reducing the dependence on a specific template structure and thus improving the accuracy rate. Moreover, hard templates, being manually designed, are susceptible to subjective biases and domain-specific assumptions, which can introduce domain bias. In contrast, soft templates are generated automatically through training processes, enabling them to more neutrally capture data characteristics and thus mitigate domain bias. Consequently, the implementation of soft templates can substantially enhance the overall performance of the model.

**Table 3:** Accuracy (%) of different templates.

**Tasks**	**Templats**				**Ours**
	**0**	**1**	**2**	**3**	
Oncology → Internal	62.15	71.10	79.63	80.20	**95.47**
Surgery → Oncology	65.05	68.97	70.73	76.60	**76.87**
Pediatric → Internal	94.62	82.42	89.82	92.17	**95.28**
Pediatric → Surgery	94.30	92.73	91.93	90.43	**94.82**

The bolded result indicates the best result.

### 4.5 Influence of hyperparameters

The configuration of hyperparameters critically influences both the performance and training efficiency of the model. This section will concentrate on the experimental investigation of two key hyperparameters: batch_size and training epochs(epochs). These parameters play a pivotal role in the training process, directly impacting the rate of convergence and the overall effectiveness of model learning.

#### 4.5.1 Influence of batch_size

**Figure 3:**
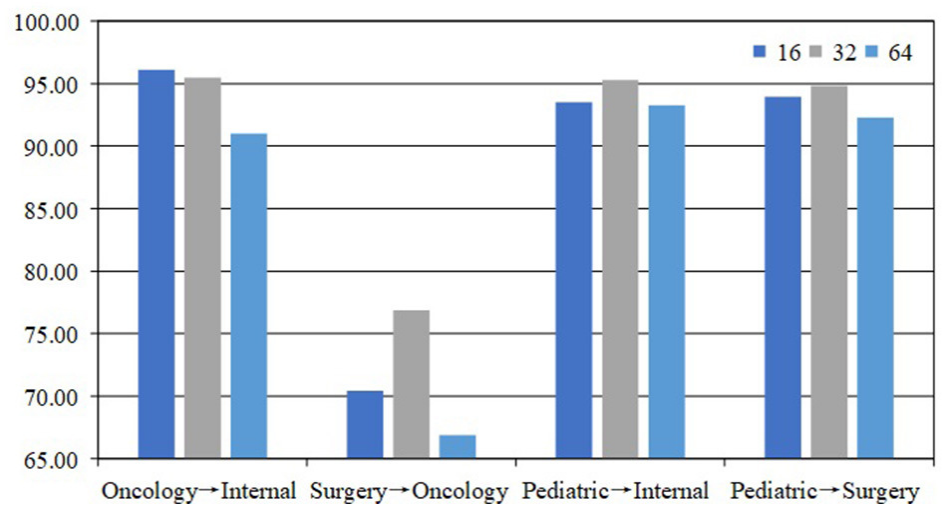
Comparison with batch_size is 16, 32, and 64 on the Medical Q&A dataset.

Batch_size is the number of samples input into the model in one iteration. It directly affects the training efficiency and memory usage of the model. We chose three batch sizes, 16, 32, and 64, for testing in our experiments, and the results are shown in Figure 3. In most tasks, the model has the most accuracy when the batch_size is 32. Our analysis suggests that an excessively large batch size may cause the model to overfit the training data, while a batch size that is too small may result in underfitting, making it difficult for the model to adequately learn the data features. Therefore, a batch_size of 32 strikes a balance, enhancing the generalization ability of the model while maintaining training stability. In addition, a batch_size that is too large may lead to memory constraints, whereas a batch_size that is too small may result in underutilization of computational resources, thus reducing training efficiency. Choosing a batch_size of 32 allows for more efficient use of hardware resources, improving training efficiency and optimizing model performance.

#### 4.5.2 Influence of epochs

**Figure 4:**
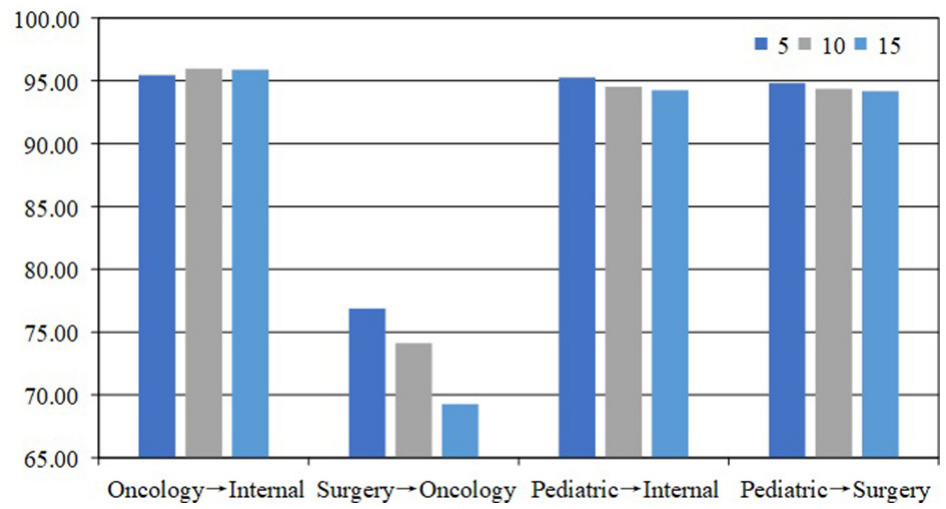
Comparison with epoch is 5, 10, and 15 on the Medical Q&A dataset.

Epoch refers to the number of times the model is trained on the entire training dataset. In our experiments, we tested three epochs: 5, 10, and 15. The results, as shown in Figure 4, indicate that the model performs best when the epoch is set to 5. Our analysis shows that fewer epochs help avoid overfitting. As the number of epochs increases, the model may overfit the training data. In addition, fewer epochs can speed up the training process and reduce training time and computational resource consumption.

## 5 Conclusion

In this study, we propose a novel medical triage method for alleviating the medical strain, which is a cross-domain text classification method based on prompt-tuning. We argue that most existing deep learning and fine-tuning PLM methods require a certain amount of labeled or unlabeled data for model training. In addition, existing approaches have not taken into account the distortion of semantic feature structure and the loss of category discriminability in the model training. To tackle the above challenges, we propose to classify patients' questions or texts about their symptoms into several given categories to give suggestions on which kind of consulting room patients could choose. The labeled source domain information is embedded into the prompt templates to generate another text with similar semantic feature structures for performing classification tasks. Experimental results show that our method outperforms the SOTA baselines for medical triage recommendation. Future work will concentrate on enhancing the prompt-tuning model and exploring more efficient methods for learning feature.

## Data Availability

The original contributions presented in the study are included in the article/supplementary material, further inquiries can be directed to the corresponding author.

## Footnotes

^1^https://github.com/Toyhom/Chinese-medical-dialogue-data.
